# Inside a duck‐billed dinosaur: Vertebral bone microstructure of *Huallasaurus* (Hadrosauridae), Upper Cretaceous of Patagonia

**DOI:** 10.1002/ar.70040

**Published:** 2025-08-28

**Authors:** Tito Aureliano, Aline M. Ghilardi, Jonatan Kaluza, Agustín G. Martinelli

**Affiliations:** ^1^ Department of Biological Chemistry, Programa de Pós‐Graduação em Diversidade Biológica e Recursos Naturais Regional University of Cariri (URCA) Crato Brazil; ^2^ Diversity, Ichnology and Osteohistology Research Group (DINOlab) Federal University of Rio Grande do Norte (URFN) Natal Brazil; ^3^ Fundación de Historia Natural “Félix de Azara” Universidad Maimónides/CONICET Ciudad Autónoma de Buenos Aires Argentina; ^4^ Sección Paleontología de Vertebrados Museo Argentino de Ciencias Naturales “Bernardino Rivadavia”‐CONICET Ciudad Autónoma de Buenos Aires Argentina

**Keywords:** CT scan, microanatomy, morphology, Ornithischia, respiratory system

## Abstract

Dinosaurs evolved a unique respiratory system with air sacs that contributed to their evolutionary success. Postcranial skeletal pneumaticity (PSP) has been used to infer the presence of air sac systems in some fossil archosaurs. While unambiguous evidence of PSP is well documented in pterosaurs and post‐Carnian saurischians, it remains absent within Ornithischia, challenging phylogenetic predictions. We used computed tomography to examine the internal vertebral microanatomy of three *Huallasaurus australis* specimens, a saurolophine hadrosaur from the Late Cretaceous of Patagonia, Argentina. The internal structure reveals a relatively dense trabecular architecture lacking evidence of invasive pneumaticity across the centra, neural arches, and neural spines, contrasting with the condition in post‐Carnian saurischians. The internal vertebral pattern of *Huallasaurus* resembles that of silesaurs more than other apneumatic archosauriforms. These observations are consistent with the hypothesis that invasive air sac diverticula did not evolve in Ornithischia and align with the previously proposed “pelvic bellows” ventilation model for the group. The internal vertebral architecture in this hadrosaur shows superficial similarities to the trabecular structure seen in some large mammals, although functional equivalence remains speculative. The absence of invasive air sacs in *Huallasaurus*, combined with dense trabecular matrix and thin cortical walls, may have supported large body sizes or accommodated intraosseous fat reserves, though this requires further testing. This stock of fatty tissues could have provided energy for these hadrosaurs during regional migration, as observed in modern migratory mammals.

## Graphical abstract

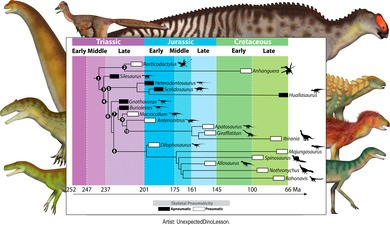

## 1 INTRODUCTION

Dinosaurs represent a successful group that survived for over 233 million years. Air sac evolution may have contributed to their success by enhancing ventilation, reducing body density, and possibly aiding thermoregulation in larger organisms (Brocklehurst et al., 2020; Maina, 2006; Perry et al., 2009; Wedel, 2009). Macroscopic traces of these invasive respiratory tissues are primarily identified through postcranial skeletal pneumaticity (PSP), characterized by well‐defined laminae and deep fossae (foramina) connected with internal pneumatic chambers (Britt, 1993; O'Connor, 2006; Wedel et al., 2000). Notably, unambiguous PSP evidence in dinosaurs remains limited to post‐Carnian saurischians (Aureliano et al., 2023, 2024; Butler et al., 2012; Cerda et al., 2012; Forster et al., 2020; Schachner et al., 2009; Smith et al., 2021; Watanabe et al., 2015; Wedel, 2003; Windholz et al., 2020; Zurriaguz, 2024), while it appears absent in ornithischians. The absence of PSP in the earliest saurischians (Aureliano et al., 2022) and its presence in pterosaurs (Butler et al., 2009) implies that the evolution of PSP occurred independently at least three times: in sauropodomorphs, theropods, and pterosaurs. Ornithischians, however, remain outliers among ornithodirans due to their lack of PSP, contradicting parsimonious inferences predicting the presence of air sacs based on phylogenetic bracketing (Butler et al., 2012). Modifications in the thoracic and pelvic regions in ornithischians, plus the presence of gastralia early on in the evolution of the clade, are other morphological adaptations suggesting a unique “pelvic bellows” ventilation system in this group (Radermacher et al., 2021). Here we describe the internal vertebral microanatomy of three individuals of *Huallasaurus australis* (Bonaparte et al., 1984; Rozadilla et al., 2022), a saurolophine hadrosaur from the Upper Cretaceous of Northern Patagonia, Argentina. Our CT scan analysis reveals a distinctive internal architecture of the axial skeleton that differs markedly from the pneumatized condition seen in saurischians. These findings offer novel insights into the evolution of skeletal and possibly respiratory adaptations in *Huallasaurus*, with broader implications for understanding the unique functional morphology of ornithischian dinosaurs.

## 2 MATERIALS AND METHODS

Institutional abbreviations: AM, Albany Museum, Makanda, South Africa; CAPPA/UFSM, Centro de Apoio à Pesquisa Paleontológica da Quarta Colônia, Universidade Federal de Santa Maria, São João do Polêsine, Rio Grande do Sul, Brazil; CEM, Laboratório de Ecologia, Federal University of Paraná, *campus* Pontal do Paraná, Brazil; MACN‐Pv, Museo Argentino de Ciencias Naturales “Bernadino Rivadavia”, Colección Nacional de Paleovertebrados, Buenos Aires, Argentina; NHMUK, Natural History Museum, London, UK; SAM, Iziko South African Museum, Cape Town, South Africa.

### 2.1 Specimens

We analyzed three specimens referable to *H. australis* (Rozadilla et al., 2022). The selected elements of MACN‐Pv RN02 include two cervical, one anterior dorsal, and one anterior caudal vertebra. The selected element of MACN‐Pv RN146 comprises one posterior dorsal vertebra. The selected elements of MACN‐Pv RN826 include one cervical, one anterior dorsal vertebra, and two posterior dorsal vertebrae. MACN‐Pv RN02 has been designated the holotype of “*Kritosaurus*” *australis* (Bonaparte et al., 1984) was described in later contributions (Bonaparte & Rougier, 1987). Subsequently, the species was designated part of the genus *Secernosaurus*, as *S. australis* (Prieto‐Marquez & Salinas, 2010). The review of all specimens from the Los Alamitos Formation permitted the proposal of a new genus, *H. australis*, with topologies recovering this taxon within saurolophine hadrosaurids, within the Kritosaurini tribe (Rozadilla et al., 2022).

### 2.2 Remarks on ontogeny

The neurocentral suture is completely fused in all specimens, suggesting at least moderate ontogenetic maturity in all individuals (Brochu, 1996; Hone et al., 2016). However, the three specimens differ in size. MACN‐Pv RN146 is considerably smaller than MACN‐Pv RN02, and the latter is smaller than MACN‐Pv RN826. Therefore, all specimens were likely individuals from different ontogenetic stages.

### 2.3 Locality and horizon

The specimens came from the Upper Cretaceous (Campanian‐Maastrichtian) Los Alamitos Formation, North Patagonian Massif, Río Negro Province, Argentina (Bonaparte et al., 1984; Prieto‐Marquez & Salinas, 2010).

### 2.4 Anatomical nomenclature

We followed Wilson (1999, 2012) and Wilson et al. (2011) nomenclature for vertebral laminae and fossae. We followed the terminology of Wedel (2003, 2007), Wedel et al. (2000), O'Connor (2006), and Aureliano et al. (2021) for the internal vertebral structures.

### 2.5 Computed tomography (CT scan)

**FIGURE 1:**
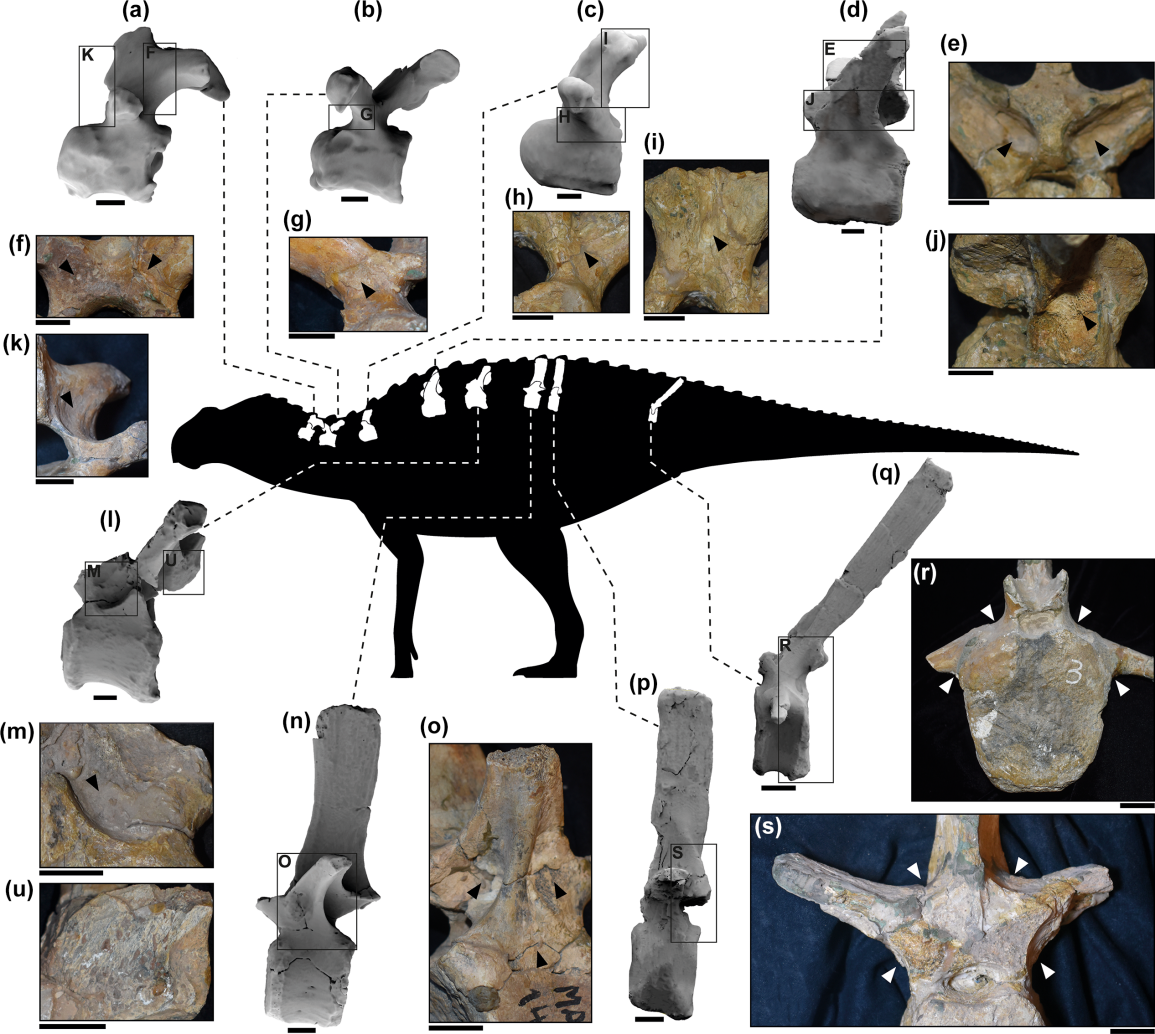
Reconstruction of the saurolophine hadrosaur *Huallasaurus australis* showing the cervical (a–c,f–i,k), dorsal (d,e,j,l–p,s,u), and caudal (q,r) vertebrae analyzed in this study. Three‐dimensional reconstructions from CT scans in (a–d,l,n,p,q). MACN‐Pv RN02.a in (a,f,k); MACN‐Pv RN02.b in (b,g); MACN‐Pv RN826.a in (c,h,i); MACN‐Pv RN826.b in (d,e,j); MACN‐Pv RN02.c in (l,m,u); MACN‐Pv RN146.a in (l,m,u); MACN‐Pv RN826.c in (p,s); MACN‐Pv RN02.d in (q,r). Photographs showing blind fossae in (e–k,m,o,r,s) (arrows). Detail of a worn portion of the neural arch exposing an internal apneumatic trabecular architecture (u). Left lateral view in (a–d,l,n,p,q). Right lateral view in (j,m,o). Caudal view in (e–i,r,s). Cranial view in K. Scale bar = 20 mm. Reconstruction is not to scale.

Vertebrae were scanned at Maimónides University (Buenos Aires, Argentina) using a Siemens‐Somatom Perspective 128‐Channel Multislice CT scanner with 0.75 mm voxel size. Acceleration voltage varied between 90 and 120 kV in a current of 367 mA. We followed Aureliano et al. (2020, 2021) to elaborate densitometry‐based rainbow color ranges based on the non‐dimensional Hounsfield counts (Figure 1). Our CT densitometry provides relative density contrasts within each scan but not absolute bone density values. Color scales reflect relative attenuation, primarily distinguishing dense cortical bone from trabecular bone and infilling matrix, but are not standardized across different specimens due to potential variation in fossilization and matrix composition. Therefore, we opted for a qualitative approach since mineral infills in bone pores and diagenetic differences between specimens hinder quantitative analyses. We used 3D Slicer version 5.2.1 for the scan analyses (Fedorov et al., 2012) and CloudCompare (Girardeau‐Montaut, 2016) for generating the 3D model reconstructions seen in Figure 1. Given the voxel resolution of 0.75 mm, fine‐scale trabecular details are visible in larger vertebral regions but may be underestimated in thinner cortical structures or small features. All interpretations are therefore conservative with respect to resolution limitations. All scans were uploaded to Morphobank and are available for download through this link: https://www.morphosource.org/projects/000698046.

## 3 RESULTS

**FIGURE 2:**
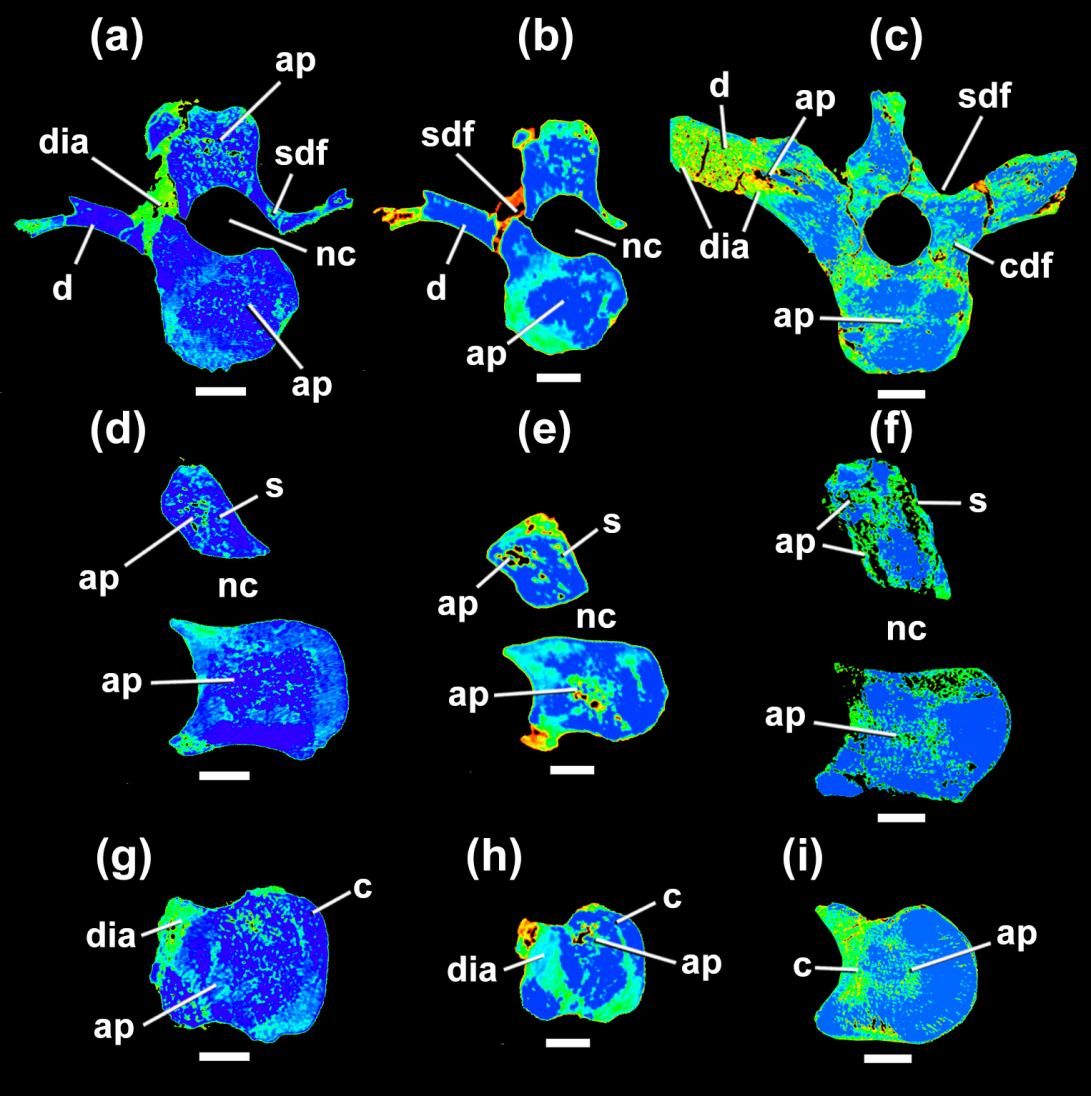
Computed tomography densitometry analysis of the hadrosaur *Huallasaurus* cervical vertebrae in transverse (a–c), parasagittal (d–f) and frontal (g–i) views. MACN‐Pv RN02.a in (a,d,g). MACN‐Pv RN02.b in (b,e,h). MACN‐Pv RN826.a in (c,f,i). Note the absence of deep fossae connecting to large internal chambers, demonstrating the lack of PSP in the elements. Lighter blue and green indicate lower densities (e.g., small apneumatic chambers). Darker blue illustrates denser structures (e.g., compact bone tissue, dense trabecular matrix etc). Note that variations may occur due to diagenetic artifacts. ap, apneumatic bone; c, centrum; cdf, centrodiapophyseal fossa; d, diapophysis; dia, diagenetic artifacts; nc, neural canal; s, neural spine; sdf, spinodiapophyseal fossa. Scale bar = 20 mm.

**FIGURE 3:**
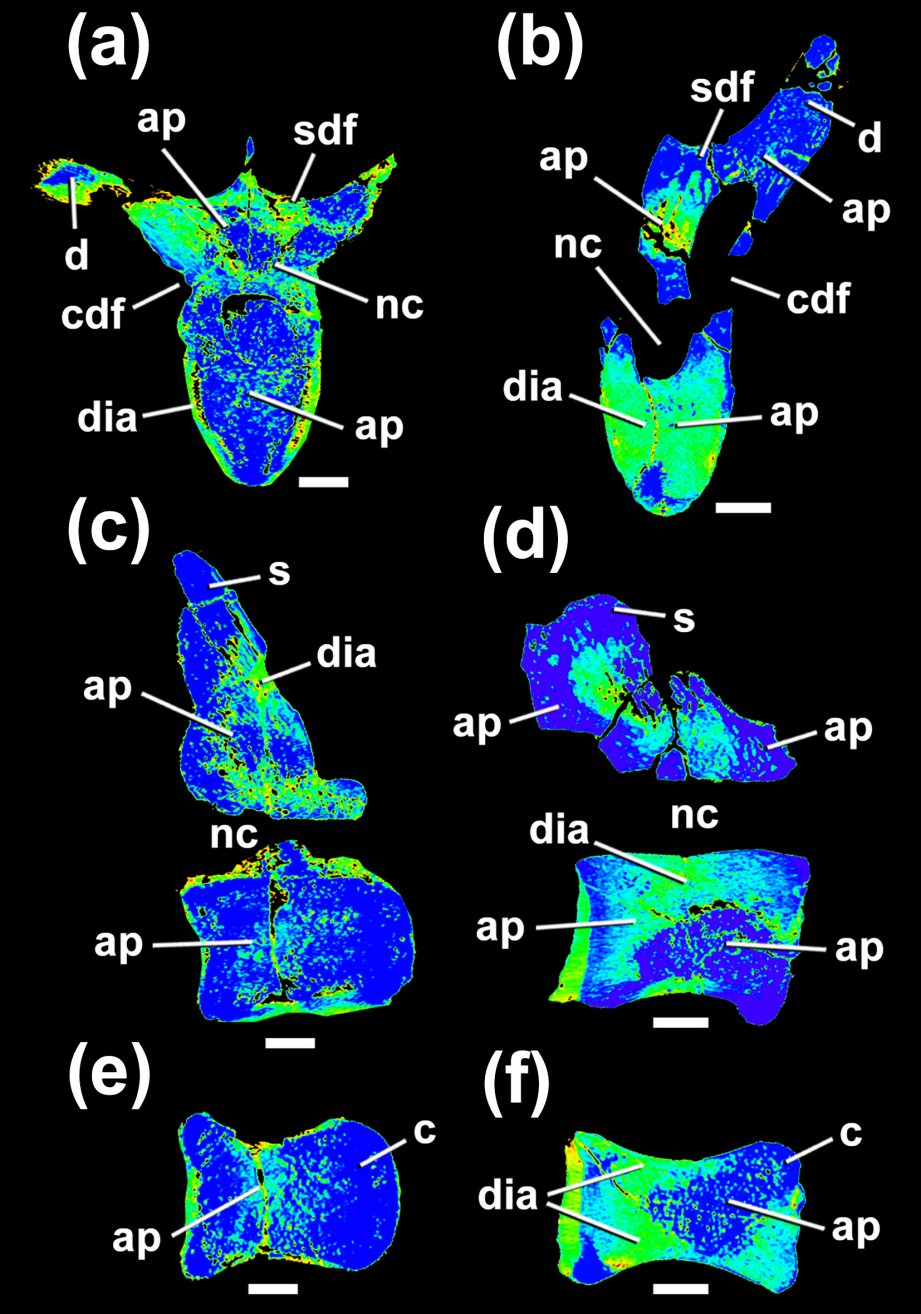
Computed tomography densitometry analysis of the hadrosaur *Huallasaurus* anterior to middle dorsal vertebrae in transverse (a,b), parasagittal (c,d) and frontal (e,f) views. MACN‐Pv RN826.b in (a,c,e). MACN‐Pv RN02.c in (b,d,f). Note the absence of deep fossae connecting to large internal chambers, demonstrating the lack of PSP in the vertebrae. Lighter blue and green indicate lower densities (e.g., small apneumatic trabeculae). Darker blue illustrates denser structures (e.g., compact bone tissue, dense trabecular matrix etc). Note that variations may occur due to diagenetic artifacts. ap, apneumatic bone; c, centrum; cdf, centrodiapophyseal fossa; d, diapophysis; dia, diagenetic artifacts; nc, neural canal; s, neural spine; sdf, spinodiapophyseal fossa. Scale bar = 20 mm.

**FIGURE 4:**
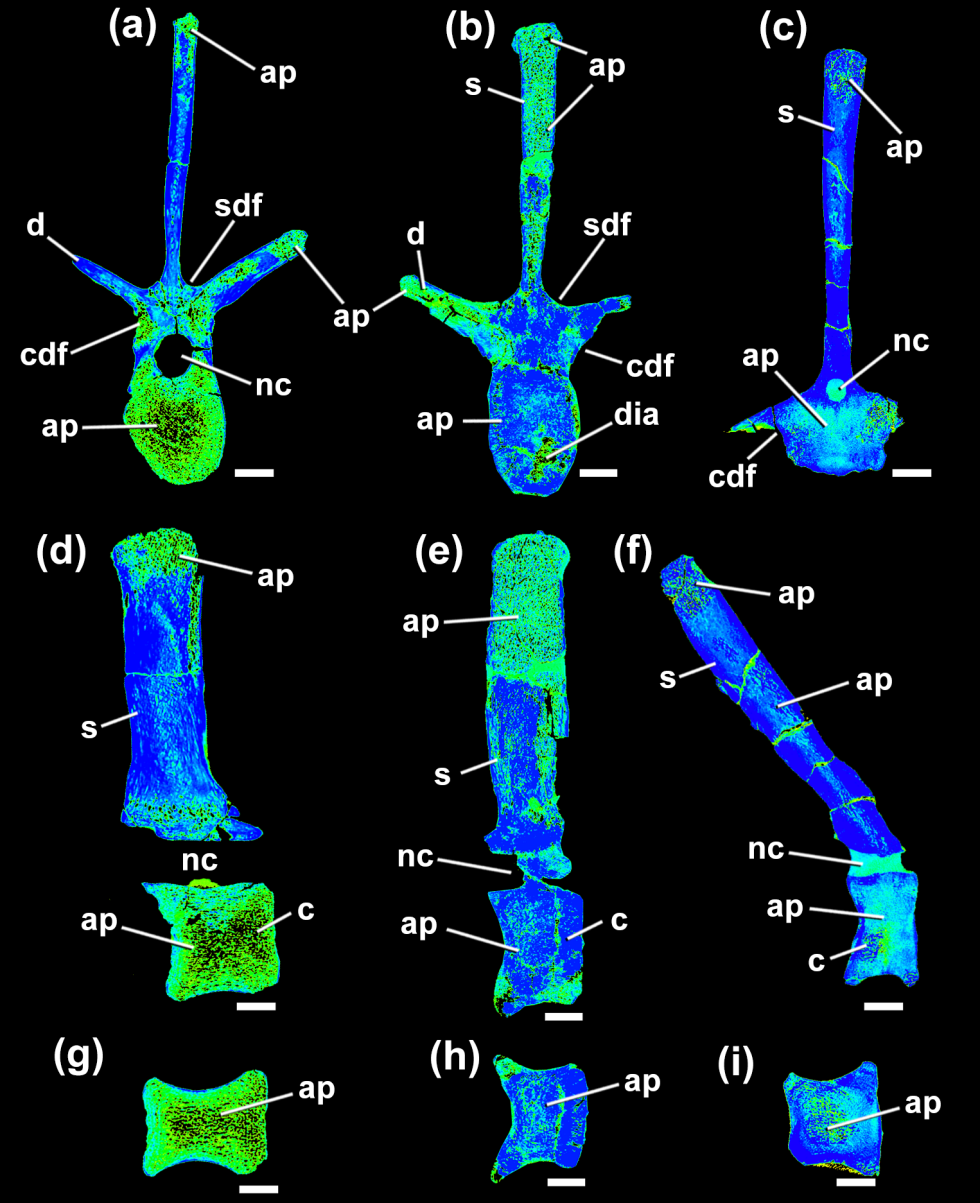
Computed tomography densitometry analysis of the hadrosaur *Huallasaurus* posterior dorsal (a,b,d,e,g,h) and caudal (c,f,i) vertebrae in transverse (a–c), parasagittal (d–f), and frontal (g–i) views. MACN‐Pv RN146.a in (a,d,g). MACN‐Pv RN826.c in (b,e,h). MACN‐Pv RN02.d in (c,f,i). Note the absence of deep fossae connecting to large internal chambers, demonstrating the lack of PSP in the vertebrae. Lighter blue and green indicate lower densities (e.g., small apneumatic trabeculae). Darker blue illustrates denser structures (e.g., compact bone tissue, dense trabecular matrix etc). Variations may occur due to diagenetic artifacts. ap, apneumatic bone; c, centrum; cdf, centrodiapophyseal fossa; d, diapophysis; dia, diagenetic artifacts; nc, neural canal; s, neural spine; sdf, spinodiapophyseal fossa. Scale bar = 20 mm.

The CT scans of *Huallasaurus* revealed the microanatomy preserved without major diagenetic artifacts (Figures 2, 3, 4), except for MCN‐PV RN 02.b and MCN‐PV RN 186.a and 02.b, in which minerals caused visual anomalies in some portions (Figures 2a,c,h and 3a–d,f). Notwithstanding, the internal architecture of all vertebrae could be visualized (Figures 2, 3, 4).

The cervical vertebrae present a few laminae with shallow blind fossae in the neural arch (Figure 1a–c,f–i,k). The centra show no lateral fossae. There are two small parallel craniocaudally elongated vascular foramina in the right lateral centrum of MACN‐Pv RN 02.b. The internal structure shows dense trabecular bone matrix without pneumatic cavities throughout the vertebra (Figure 2).

The anterior to middle dorsal vertebrae present laminae with slightly deeper centrodiapophyseal fossae compared with the cervicals (Figure 1d,l). Spinodiapophyseal fossae are very shallow. However, all fossae are blind (Figure 1e,j,m). The centra present no lateral fossae. A fortuitous break exposes a dense trabecular array inside the neural arch of MACN‐Pv RN02 (Figure 1u). The internal structure comprises a trabecular bone matrix of moderate density, without evidence of pneumatic chambers (Figure 3). However, there is a noticeable decrease in trabecular density in the areas under the spinodiapophyseal fossae and the neural spine, compared to the cervical vertebrae (Figure 3a–d).

The posterior‐most dorsal vertebra MACN‐Pv RN146.a presents laminae with slightly deep centrodiapophyseal fossae as observed in the anterior to middle dorsals (Figure 1n,o). The posterior‐most dorsal vertebra MACN‐Pv RN826.c and the anterior caudal MACN‐Pv RN02.d present shallow to nearly absent centrodiapophyseal and spinodiapophyseal fossae (Figure 1p–s). Nevertheless, all fossae are still blind (Figures 1n–s and 4). The centra of MACN‐Pv RN146.a and MACN‐Pv RN02.d present no lateral fossae. There are three small parallel craniocaudally elongated lateral vascular foramina in the centrum of MACN‐Pv RN826.c. Internally, the vertebrae exhibit an apneumatic trabecular matrix that remains relatively dense across the centra and neural arches, with no evidence of internal pneumatic cavities (Figure 4). The tall neural spines present a thick cortical architecture in most of their extension (Figure 4a–f). Cortical walls are reduced in the centra and in the extremities of the diaphyses and the neural spines in both the posterior‐most dorsals and the anterior caudal.

## 4 DISCUSSION

### 4.1 Evolution of the dinosauriform vertebral microanatomy

**FIGURE 5:**
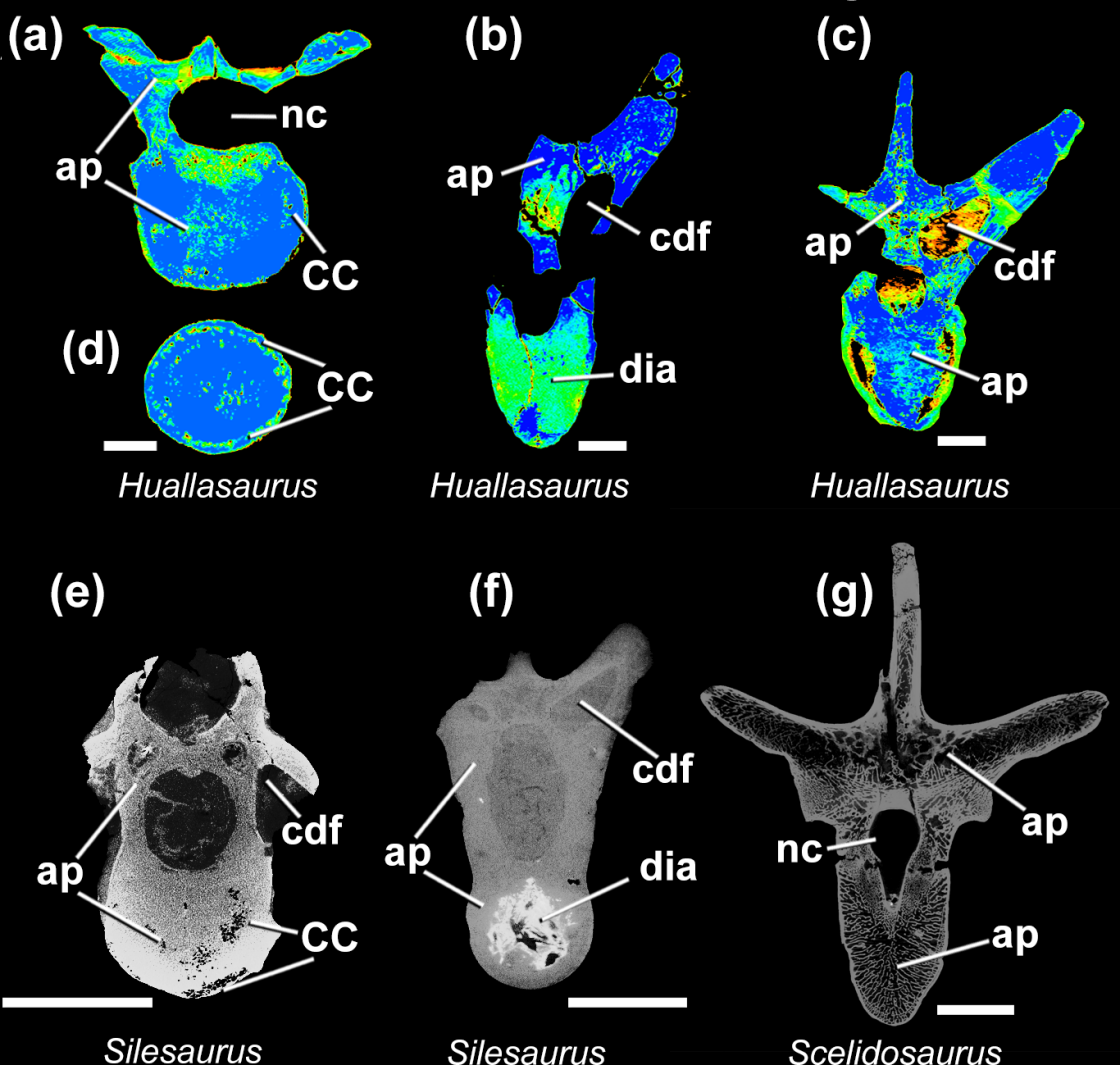
CT scan slices comparison between the vertebrae of ornithischians and *Silesaurus*. The hadrosaur *Huallasaurus* MACN‐Pv RN826.a (a,d); MACN‐Pv RN826.b (b); MACN‐Pv RN 02.c (c). *Silesaurus* ZPAL Ab III 1299 (e); ZPAL Ab III 423/6 (f) (Butler et al., 2012). (g) The thyreophoran *Scelidosaurus* NHMUK R1111 (Butler et al., 2012). Cervical vertebrae in (a–c). Dorsal vertebrae in (b,c,f,g). All in transverse view. ap, apneumatic bone; cc, circumferential chambers; cdf, centrodiapophyseal fossa; dia, diagenetic artifacts; nc, neural canal. Scale bar in a–d,g = 20 mm; in e,f = 10 mm.

Our results show no evidence of internal pneumatic structures in *Huallasaurus* vertebrae. The overall density of the trabecular array is less compact than in the centra of the archosauriforms *Stenaulorhynchus* NHMUK R36618, *Erythrosuchus* NHMUK R3592, and the Phytosauria indet. NHMUK OR38072 (Butler et al., 2012). However, the vertebrae of *Huallasaurus* were denser than in the herrerasaurid *Gnathovorax* CAPPA/UFSM 0035 and in the early sauropodomorphs *Buriolestes* CAPPA/UFSM 0035 and *Pampadromaeus* ULBRA‐PV016 (Aureliano et al., 2022). Also, the vertebrae were less vascularized than the condition seen in the early thyreophoran *Scelidosaurus* NHMUK R1111, especially the caudals (Butler et al., 2012). Interestingly, the internal apneumatic architecture of *Huallasaurus* appears superficially similar to that observed in the silesaurid ZPAL Ab III vertebral series (Butler et al., 2012), sharing even the tiny centripetal circumferential chambers and the vascularized clusters surrounding the cotyles (Figure 5).

Variation in trabecular architecture across cervical, dorsal, and caudal vertebrae may reflect biomechanical loading or positional anatomy differences rather than evolutionary signals alone. Furthermore, given the different sizes of the specimens, potential ontogenetic effects cannot be ruled out.

### 4.2 Evolution of the ornithischian respiration

Anatomical evidence from *Heterodontosaurus tucki* (AM 4766 and SAM‐PK‐K1332) suggested the “pelvic bellows” model for ornithischian ventilation (Radermacher et al., 2021). *Heterodontosaurus tucki* exhibits reduced gastralia, an elaborate sternum, mobile sternal ribs, and an incipient anterior pubic process (APP), indicating a transition from cuirassal breathing (Radermacher et al., 2021). Early ornithischians likely used a “puboperitoneal muscle” attached to the APP as an accessory ventilator. Later genasaurians (i.e., hadrosaurs) lost gastralia, and APP elongation suggested this muscle became the primary ventilator. Vertebrocostal orientations indicated a bipartite, dorsally immobile lung, unlike the crocodilian hepatic piston, but convergent in pelvic involvement, with the puboperitoneal muscle acting on flexible lung regions.

The vertebral morphology of ornithischian dinosaurs provides additional support for this respiratory model. Analysis of costovertebral joint anatomy across archosaurs reveals that ornithischians, like other non‐avialan dinosaurs, possessed separate parapophyses and diapophyses throughout their dorsal vertebral series, creating a furrowed thoracic ceiling similar to that of modern birds (Brocklehurst et al., 2020; Schachner et al., 2011). This contrasts with the smooth thoracic ceiling of crocodilians, where parapophyses migrate onto transverse processes, allowing greater lung mobility during hepatic piston ventilation (Brocklehurst et al., 2020). The furrowed thoracic ceiling in ornithischians would have provided structural support for dorsally immobile, heterogeneously partitioned lungs with a rigid dorsocranial region containing the majority of gas‐exchange tissue, ventilated by a more flexible and less vascularized caudal region (Schachner et al., 2011).

The evolution of ornithischian respiratory mechanics likely involved a transition from the ancestral cuirassal breathing mechanism proposed for basal archosaurs, which utilized the gastralial basket and ischiotruncus muscle for ventilation (Schachner et al., 2011), to the specialized pelvic bellows system. This transition is evidenced by the progressive reduction and eventual loss of gastralia in more derived ornithischians, coupled with the development of the APP and associated musculature (Radermacher et al., 2021).

**FIGURE 6:**
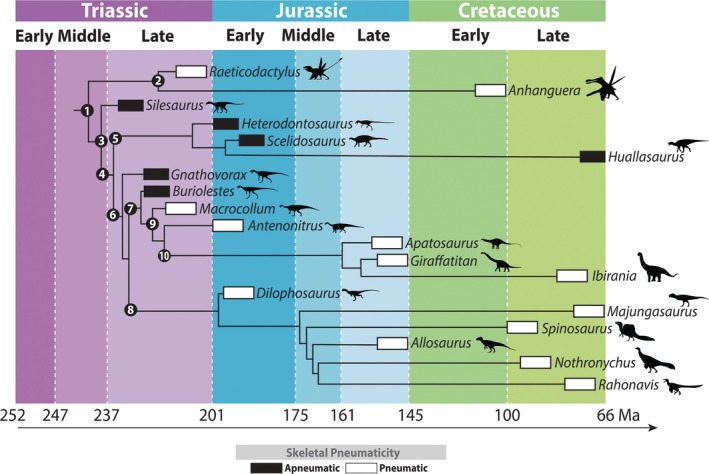
Chronological and phylogenetic evolution of postcranial skeletal pneumaticity in ornithodirans. 1, Ornithodira; 2, Pterosauria; 3, Dinosauromorpha; 4, Dinosauria; 5, Ornithischia; 6, Saurischia; 7, Sauropodomorpha; 8, Theropoda; 9, Plateosauria; 10, Sauropoda. *Allosaurus* (Smith et al., 2021), *Apatosaurus* (Wedel & Taylor, 2013), *Anhanguera* (Buchmann et al., 2021), *Antenonitrus* (Yates et al., 2012), *Buriolestes* (Aureliano et al., 2022), *Dilophosaurus* (Marsh & Rowe, 2020), *Giraffatitan* (Wedel & Taylor, 2013), *Gnathovorax* (Aureliano et al., 2022), *Heterodontosaurus* (Radermacher et al., 2021), *Ibirania* (Aureliano et al., 2021), *Macrocollum* (Aureliano et al., 2023), *Majungasaurus* (Aureliano et al., 2024; O'Connor, 2006), *Nothronychus* (Smith et al., 2021), *Raeticodactylus* (Butler et al., 2009), *Rahonavis* (Aureliano et al., 2024; Forster et al., 2020), *Scelidosaurus* (Butler et al., 2012), *Silesaurus* (Butler et al., 2012), and *Spinosaurus* (Myhrvold et al., 2024).(Aureliano et al., 2024; Forster et al., 2020). Silhouettes are from Phylopic.org by Alessio Ciaffi, Cy Marchant, Iain Reid, Jaime Headden, JF Designs, Julio Garza, Mathew Wedel, Matthew Dempsey, Tasman Dixon, Walter Vladimir, and Will Toosey. Adapted from Aureliano et al. (2023).

Our observations in *Huallasaurus* are consistent with the hypothesis that invasive air sac diverticula did not evolve in Ornithischia, in which the apneumatic postcranial skeleton remained a plesiomorphic adaptation inherited from their Triassic ancestors. Our finding is compatible with the “pelvic bellows” model for ornithischian ventilation (Radermacher et al., 2021) and with previous evidence suggesting multiple origins (Figure 6) for the evolution of the PSP in ornithodirans (Aureliano et al., 2022, 2023; Butler et al., 2012).

### 4.3 Remarks on dinosaur systematics

Some studies suggested a paraphyletic condition for silesaurs, recovering them as early‐diverging ornithischians (Cabreira et al., 2016; Cau, 2024; Müller & Garcia, 2020; Norman et al., 2022). This hypothesis also finds support in morphological data, since the PSP is present in post‐Carnian saurischians but seemingly absent in ornithischians, based on both macroscopic observations and tomography of Jurassic taxa *Heterodontosaurus* (Radermacher et al., 2021) and *Scelidosaurus* (Butler et al., 2012). Our results now suggest that *Huallasaurus*, and possibly other hadrosauriforms, did not only retain an internal vertebral microanatomy similar to Triassic archosauriforms, but shared the vascular pattern with *Silesaurus* more than with any other archosauriforms (Figure 6). Therefore, our results may contribute to the ongoing discussion regarding the paraphyletic condition of early dinosaurs, particularly with respect to internal vertebral microanatomy, although this should be tested further with broader sampling.

### 4.4 Remarks on hadrosaur vertebral morphology

**FIGURE 7:**
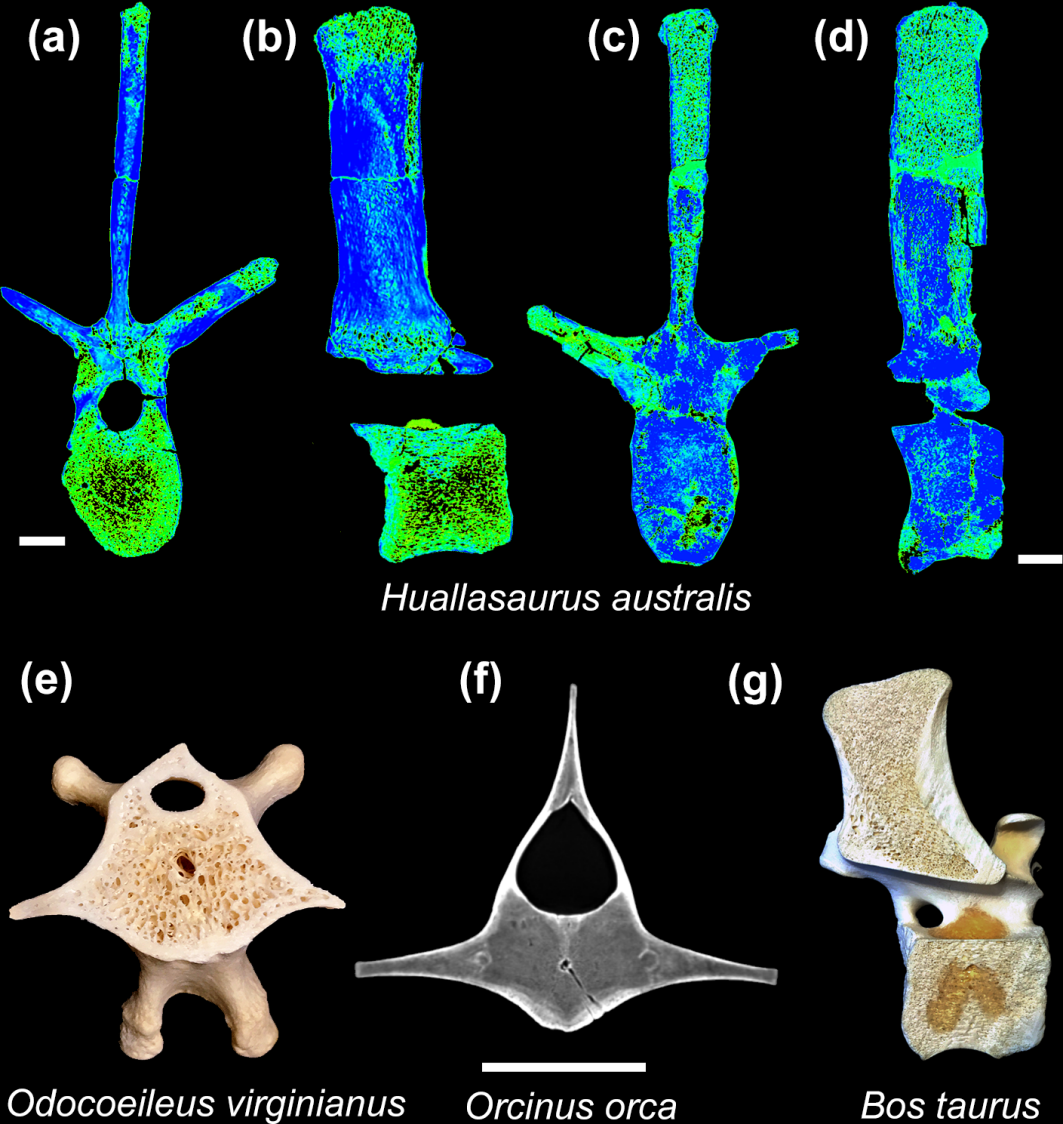
Comparison between the dense trabecular tissue in the vertebrae of the hadrosaur *Huallasaurus australis* (a–d) and the internal structures in modern mammals (e–g). (e) Bone cross section of *Odocoeileus virginianus*, a North American deer (unnumbered; courtesy of Mathew Wedel). (f) CT scan of *Orcinus orca*, the orca whale (lumbar; CEM 038977; courtesy of Gabriel L. Gomes). (g) Bone cross section of *Bos taurus*, the domestic bull (unnumbered; courtesy of Mathew Wedel). Transverse view in (a, c, e, f). Parasagittal view in (b,d,g). Scale bar in (a–d) = 20 mm; in (f) = 10 mm. (e,g) Not to scale.

The analyses of *Huallasaurus* vertebrae reveal a trabecular bone arrangement that appears denser than that observed in several other dinosaur taxa examined in previous studies, although this may reflect biomechanical adaptations, taxon‐specific factors, or ontogenetic stages. The presence of a dense trabecular matrix and the absence of large internal chambers throughout the vertebral series represent a condition that, intriguingly, shows superficial similarity to that observed in large mammals, including the domestic bull (*Bos taurus*), the North American deer (*Odocoeileus virginianus*), and the orca whale (*Orcinus orca*) (Figure 7). Likewise, the extreme reduction of cortical walls in the centra and certain regions of the neural spines mirrors a condition seen in aquatic mammals such as cetaceans (Buffrénil & Schoevaert, 1988).

This reduction in total cortical bone volume, paired with the expansion of a lightweight yet dense trabecular matrix, reflects an architecture that decreases body density while maintaining structural integrity (Fabbri et al., 2022; Houssaye et al., 2015, 2016; Myhrvold et al., 2024). In extant mammals, such trabecular spaces are frequently occupied by marrow tissues, including fat and hematopoietic cells, functioning as intraosseous energy reserves (Butler et al., 2012; O'Connor, 2006; Prothero, 1995). The absence of PSP in *Huallasaurus*, and most likely in other hadrosaurs, added to this dense internal trabecular matrix, aiding these organisms in the evolution of large body sizes and extra stocks of fat. While we cannot directly confirm the presence of such fatty deposits in *Huallasaurus*, it is plausible that the dense trabecular network could have supported similar metabolic functions, particularly given isotopic evidence suggesting regional migratory behaviors in hadrosaurs (Terrill et al., 2020), as observed in modern migratory mammals (Prothero, 1995).

Importantly, this hypothesis remains speculative and requires further testing through broader sampling of hadrosaur vertebral microanatomy, ideally incorporating histological sections to directly assess bone marrow space and tissue composition. Nevertheless, our observations provide a compelling foundation for considering how skeletal architecture may have interacted with ecological and physiological demands in large‐bodied ornithischians.

### 4.5 Vertebral internal structure and body size evolution in dinosaurs

The remarkable evolutionary success of hadrosaurs in achieving large body sizes without PSP provides compelling evidence that extensive pneumatization represents an advantageous rather than essential adaptation for dinosaur gigantism. *Shantungosaurus giganteus*, reaching 16 m in length and weighing 15 metric tons, represents one of the largest terrestrial herbivores ever recorded, demonstrating that alternative skeletal architectures could support extreme body sizes without the respiratory adaptations that characterized saurischian giants (Hu, 1973; Xu et al., 2000). The dense trabecular matrix observed in hadrosaur vertebrae likely represents a biomechanical solution to the structural challenges of large body size in the absence of pneumatic weight reduction, paralleling the trabecular architecture seen in large mammals where trabecular density increases rather than trabecular thickness as body size scales up (Aguirre et al., 2020; Barak et al., 2013). While sauropods achieved gigantism through extensive PSP that reduced body density while maintaining structural integrity, hadrosaurs evolved a different strategy, utilizing dense, apneumatic trabecular architecture (Aureliano et al., 2021; Butler et al., 2012; Cerda et al., 2012; Wedel, 2003). The comparative body size data support this hypothesis, as moderately large dinosaurs like *Tyrannosaurus rex* achieved comparable dimensions to the largest hadrosaurs despite possessing extensive pneumatization, whereas the truly gigantic sauropods like Argentinosaurus, reaching lengths of over 30 m and masses exceeding 30 tons, likely required the extreme body density reduction provided by extensive pneumatization to achieve such unprecedented sizes (Campione & Evans, 2012; D'Emic, 2023). This suggests a threshold effect where PSP becomes increasingly critical as body size approaches the upper limits of terrestrial vertebrate physiology, with multiple evolutionary pathways leading to dinosaur gigantism representing different solutions to the biomechanical challenges of large body size.

### 4.6 Limitations and future directions

We acknowledge that our discussions are based on a limited number of vertebrae from three individuals of varying sizes and uncertain ontogenetic stages. Additionally, differences in vertebral region (cervical, dorsal, caudal) and potential taphonomic alterations may influence trabecular architecture. The medical‐grade CT (0.75 mm voxels) allowed broad qualitative assessments but lacked the resolution necessary to quantify cortical thickness, trabecular spacing, and the precise volume of any trabecular cavities. Future work using high‐resolution micro‐CT (≤50 μm voxels) will enable voxel‐based morphometry of these parameters, particularly in delicate neural spine apices and centrum cortices. Likewise, histological thin sections cut through the centrum and neural arch could directly reveal marrow composition, secondary osteon density, and possible growth lines, providing a tissue‐level test of the fat‐storage hypothesis.

## 5 CONCLUSION

The microanatomical analysis of the vertebrae of *H. australis* provides valuable insights into the evolution of skeletal structure in ornithischian dinosaurs. The internal architecture of the axial skeleton exhibits an apneumatic trabecular matrix distributed throughout the centrum and neural arch, without signs of internal pneumatic chambers, contrasting sharply with the pneumatized condition characteristic of post‐Carnian saurischians.

Our observations reveal that the vertebral vascular pattern in *Huallasaurus* bears a notable resemblance to that of *Silesaurus*. Whether this similarity reflects deep phylogenetic relationships or convergent functional adaptations remains an open question that warrants further investigation.

Additionally, the expanded trabecular matrix coupled with remarkably thin cortical walls observed in *Huallasaurus* shows superficial similarity to the vertebral architecture of large modern mammals, which often accommodate intraosseous fat deposits. While the functional implications of this condition in *Huallasaurus* remain speculative, it may represent a biomechanical or physiological adaptation associated with supporting large body size in an apneumatic skeleton.

Given the limited sample size, the inclusion of vertebrae from different anatomical regions, and uncertain ontogenetic stages of the specimens, these conclusions should be regarded as preliminary. Nonetheless, this study highlights the potential for vertebral microanatomy to inform broader questions about respiratory evolution, body mass adaptations, and phylogenetic relationships in ornithischians. Future research incorporating histological sections, higher‐resolution imaging, and a wider taxonomic sampling across Ornithischia and Silesauridae will be crucial for testing the patterns and hypotheses proposed herein.

## AUTHOR CONTRIBUTIONS

**Tito Aureliano:** Conceptualization; investigation; funding acquisition; writing – original draft; methodology; validation; visualization; writing – review and editing; software; formal analysis; project administration. **Aline M. Ghilardi:** Conceptualization; funding acquisition; investigation; methodology; validation; writing – review and editing; supervision; formal analysis. **Jonatan Kaluza:** Investigation; visualization; formal analysis; software; funding acquisition; validation. **Agustín G. Martinelli:** Data curation; supervision; resources; funding acquisition; investigation; methodology; formal analysis; writing – original draft; writing – review and editing; validation.

## CONFLICT OF INTEREST STATEMENT

The authors declare no competing interests.

## Data Availability

The specimen is housed in a public research institution and can be accessed upon request to the collection's curator. Tomography data are available in Morphosource (links provided in Materials and Method).
